# Cyclic tangential flow filtration system for isolation of extracellular vesicles

**DOI:** 10.1063/5.0037768

**Published:** 2021-02-10

**Authors:** Kimin Kim, Jungjae Park, Jik-Han Jung, Ruri Lee, Ji-Ho Park, Jong Min Yuk, Hyundoo Hwang, Ju Hun Yeon

**Affiliations:** 1 Department of Integrative Biosciences, University of Brain Education, Cheonan 31228, Republic of Korea; 2 Department of Materials Science and Engineering, Korea Advanced Institute of Science and Technology, Daejeon 34051, Republic of Korea; 3 Department of Bio and Brain Engineering, Korea Advanced Institute of Science and Technology, Daejeon 34051, Republic of Korea; 4 BBB Inc., Seoul 05637, Republic of Korea

## Abstract

Size-based filtration techniques have been developed for high-throughput isolation of extracellular vesicles (EVs). Conventional direct filtration systems have limitations in that large particles generally not only block the pores of the membrane but also damage the particles because of the high fluid pressure. Here, we propose a cyclic tangential flow filtration (TFF) system that includes two membranes with pore sizes of 200 and 30 nm, connected to a peristaltic pump that feeds the stream flowing to the membrane for continuous circulation. The cyclic TFF system is better able to isolate the specific 30–200 nm size range in one step through dual cyclic filtration compared with direct filtration (DF) and single cyclic TFF (scTFF). We further introduced a buffer-exchange process to the dcTFF system after filtration to remove contaminants for more efficient purification. As a result of comparative evaluation of dcTFF and ExoQuick, EVs isolated by dcTFF had more abundant exosome markers and active EVs. The cyclic TFF system not only has great potential to separate EVs with high selectivity and separation efficiency in small volumes of samples but can also be used in clinical applications, including medical diagnostic procedures.

## INTRODUCTION

Exosomes are nanosized extracellular vesicles (EVs) that are involved in inter-cellular communication processes, transferring cargos originating in one cell to other cells.^1,2^ The physical properties of EVs reflect their nano-sized dimensions and cargoes of bioactive compounds.^3^ EVs are able to cross several biological barriers, such as the cell membrane, and are involved in many pathological processes.^4^ For these reasons, EVs have promising applications as drug targets, therapeutics, diagnostic biomarkers, and drug-delivery systems.^3,5^ A major challenge in the use of EVs for medical applications is the isolation process, reflecting the fact that EVs of interest are present in all types of biological fluids, which are complex and composed of heterogeneous EVs.^6,7^ Additionally, obtaining sufficient quantities of EVs for diagnosis of diseases may require the concentration and isolation of EVs to high purity.^8,9^ EVs in body fluids have emerged as promising biomarkers for diagnosis and prognosis of cancers.^10^ However, the lack of standard tools for isolating intact EVs in high yield and high purity poses a significant roadblock to the implementation of EVs for biomarker discovery.^11,12^

Conventional direct filtration systems have been widely used for various bioprocesses.^13^ In direct filtration processes, fluid flow is applied perpendicularly to the membrane, which causes accumulation of targeted particles and filter clogging issues, reducing the number of open pores and changing the hydrodynamic resistance unpredictably.^14^ This results in trapped particles between filters being squeezed; in the case of cells, the shear stresses may result in rupture, lowering isolation efficiency.^15^ Tangential flow filtration (TFF) systems have emerged as a commercially viable approach for preventing clogging problems.^16–18^ However, conventional TFF systems are implemented as a single isolation unit with one type of membrane.^3^ Isolating a specific range of nanoparticles requires higher-level filtration performance. In addition, in order to commercialize the TFF system for therapeutic applications, it is necessary to isolate and concentrate EVs from a small volume of samples.^19^ Indeed, recent studies have reported the development of more complicated platforms, such as dual-filtration approaches, but simultaneous dual continuous flow conditions are difficult to maintain for entire processes.^20,21^ Common isolation techniques for the separation of EVs include ultracentrifugation, immunoaffinity capture, and precipitation.^22,23^ However, these protocols must take into account additional steps, such as purification, concentration, and additional equipment costs, and may result in losses in exosome yield.^24,25^

Here, we present an improved EV isolation technique, called a dual-cyclic TFF (dcTFF), in which the fluid flows parallel to the surface of the membrane. Particles separated by the filters are not clogged but concentrated by being continuously transferred along one side of the filter. In this size-exclusion–based system, two polycarbonate membranes with pore sizes of 200 and 30 nm are assembled to form three chambers—a sample chamber, an isolation chamber, and a waste chamber. The dcTFF system not only reduces clogging issues but also improves isolation efficiency in terms of recovery rate and EV yield. Because the dcTFF chip consists of two modules with different membrane pore sizes, it allows simultaneous separation of EVs with a specific size range corresponding to upper and lower pore sizes, in this case 30–200 nm.

We first demonstrated the isolation efficiency of the dcTFF chip compared with direct filtration and a single-cyclic TFF (scTFF) chip. Then, we showed that dcTFF is a highly modular tool for size-based EV sorting of heterogeneous EV populations from culture media. Furthermore, we compared the EV size and morphology and evaluated two exosome biomarkers collected from outlet ports. Our platform can process multiple types of complex biofluids and, therefore, has broad applicability to early diagnosis of cancers and other diseases.

## RESULTS AND DISCUSSION

### Working principle of dcTFF

**FIG. 1.**
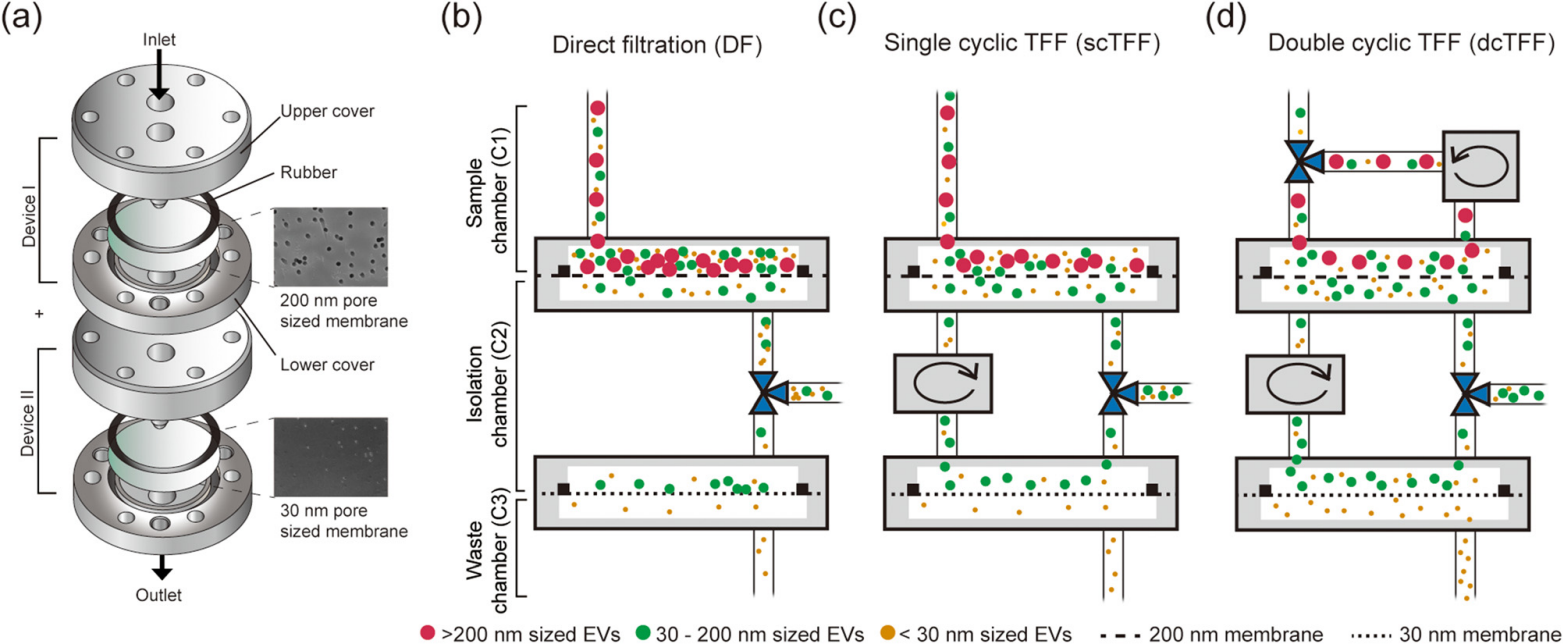
Schematics of filtration systems for isolation of EVs. (a) Schematic showing the design of the cyclic tangential filtration system, with SEM images of 200 nm and 30 nm pore sized membranes. (b)–(d) Schematic illustration of three difference methods for isolating different sizes of fluorescent beads using particles larger than the 200 nm (red), particles smaller than 200 nm but larger than 30 nm (green), and particles smaller than 30 nm (yellow): (b) direct filtration (DF), (c) single-cyclic filtration (scTFF), and (d) dual-cyclic TFF (dcTFF).

A dual-cyclic TFF (dcTFF) chip was specifically designed to allow the isolation of highly purified, concentrated exosomes [Fig. 1(a)]. We first compared isolation efficiency of three different device assemblies—direct filtration (DF), single cyclic TFF (scTFF), and dual cyclic TFF (dcTFF)—using three different kinds of fluorescent polystyrene beads (PSBs). As shown in Fig. 1(b), to allow collection of isolated samples in the C2 chamber in the assembled DF device, we added a 3-way valve between the filtration chip containing 200- and 30 nm membranes. For the assembled scTFF device, a peristaltic pump was connected between the dual-filtration chips to form a cyclic tangential flow filtration system in the DF state [Fig. 1(c)]. For the assembled dcTFF device, an additional peristaltic pump was connected to the sample chamber (C1) to form a dual-cyclic tangential flow filtration system in the scTFF state [Fig. 1(d)]. Isolation efficiency using dcTFF was determined by modeling three different behaviors using particles larger than the 200 nm (red), particles smaller than 200 nm but larger than 30 nm (green), and particles smaller than 30 nm (yellow): (1) movement of particles larger than the 200 nm filter size (red), which are continuously moved along the tubing by the peristaltic pump, thereby preventing clogging of particles in the sample chamber; (2) movement of particles smaller than 200 nm but larger than 30 nm (green), which enter the isolation chamber and are moved along the tubing by the peristaltic pump, preventing accumulation of particles on the 30 nm filter; and (3) movement of particles smaller than 30 nm (yellow), which pass through into the waste chamber.

To assess the flow rate of the dual-cyclic filtration chip, we varied the infused flow rate through the syringe pump and circulated flow rate through the peristaltic pump. Initially, when operating at an infused flow rate of 5 *μ*l/min, the device filled with air, reflecting the fact that the infused flow rate may be less than the outflow to the waste outlet. When operating at an infused flow rate of 20 *μ*l/min, disassembly occurred between the filtration chips and tubing owing to the pressure applied to the membrane.

On the basis of these results, we selected an intermediate infused flow rate of 10 *μ*l/min. Circulation flow rates of 27, 40, and 80 *μ*l/min were then assessed by measuring the extracted filtrate volume via C3. We hypothesized that the efficiency of concentration would be higher for a device in which outflow to exit waste (C3) was higher. Figure S1 shows results at a flow rate of 27 *μ*l/min, measured with a maximum amount of filtrate permeating to outlet C3. However, this flow rate was difficult to circulate against the infused flow rate of 10 *μ*l/min, a problem that was overcome by increasing the circulated flow rate to 40 *μ*l/min. On the basis of these results, we used a flow rate of 10 *μ*l/min for infusion through the syringe pump and 40 *μ*l/min for circulation through the peristaltic pump [Fig. S1(c)].

We estimated pressure drop through a porous membrane based on the Hagen-Poiseuille equation.^24^ The pressure drop is estimated by

$$\Delta P=\frac{8\mu tQ}{N\pi a^{4}},$$

where *μ* is the fluid viscosity, t is the thickness of the membrane, Q is the flow rate, N is the number of pores, and a is pore diameter. The pressure drop is estimated to be 0.04 kPa for the 200 nm membrane and 104.41 kPa for the 30 nm membrane. The pressure applied in dcTFF is about 527 times lower than ultracentrifugation and 1.4 times lower than the previous filtration system.^24^ However, experimentally, when injected at flow rates above 20 *μ*l/min, a separation occurred between the device and the tube due to the pressure applied to the small pre-sized membrane. We need to build up a module to withstand higher flow rates, such as designing a luer-lock system that connects modular units and increasing the surface area of the small pore sized membrane. In the future, we would calculate percent purity and isolation efficiency according to various flow rates through the robustness of the modular system.

### Characterization of a cyclic TFF device using fluorescent beads

**FIG. 2.**
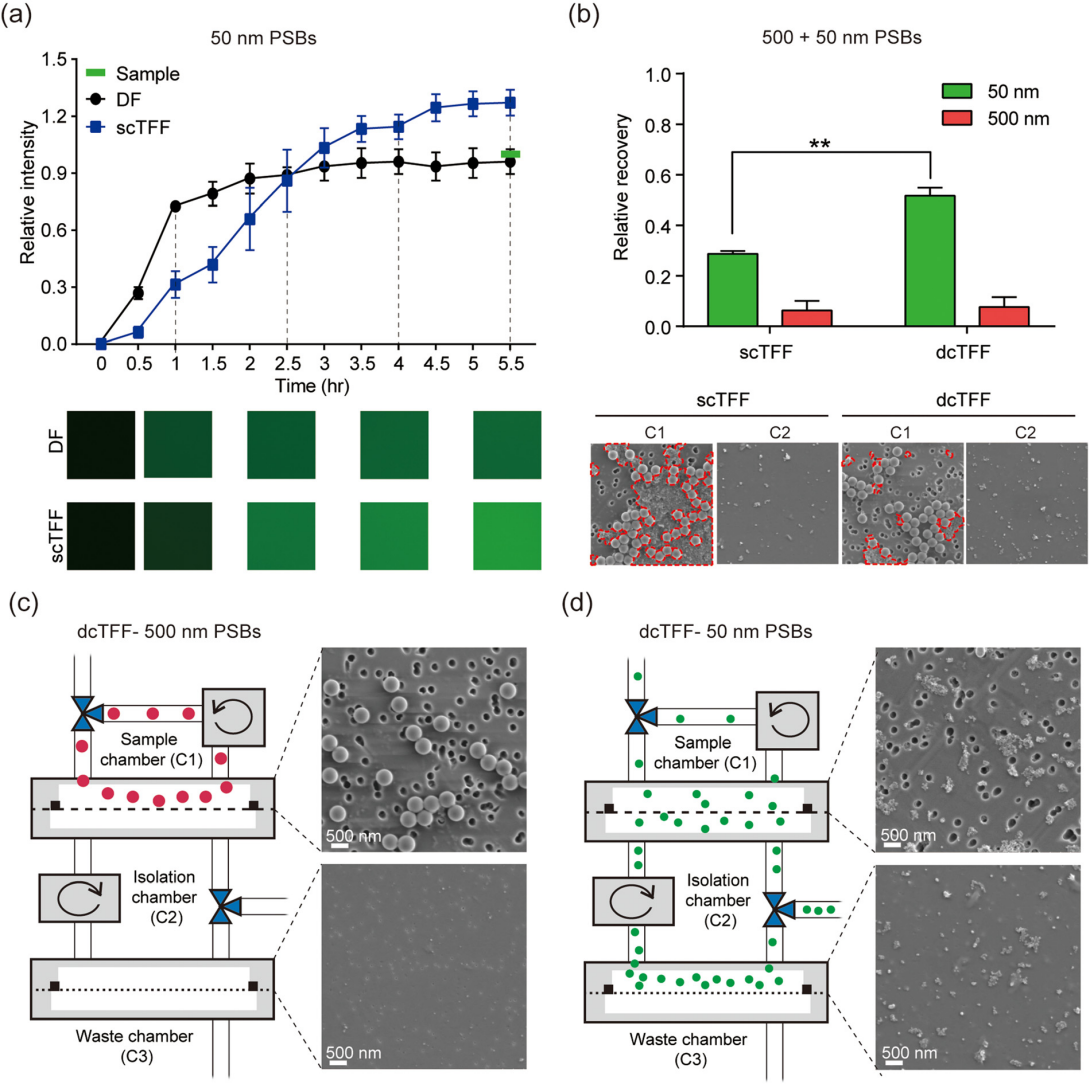
Evaluation of filtration systems using fluorescent beads. (a) Comparison of the relative intensity of fluorescent beads isolated by DF and scTFF. (b) Comparison of the relative recovery and SEM images of fluorescent beads after scTFF and dcTFF filtration. The red dotted lines represent the aggregated 50 nm PSBs. When flowed with scTFF than dcTFF, more 50 nm beads were aggregated to block the 200 nm membrane. (c) and (d) SEM images of isolated 500 nm (c) and 50 nm (d) PSBs after dcTFF filtration. Data are presented as means ± standard error of mean (SEM) (^**^ *p *<* *0.01).

To compare DF and scTFF, we measured the efficiency of particle separation using 50 nm fluorescent polystyrene beads (PSBs). Filtered 50 nm PSBs in the isolation chamber (C2) were sampled every 30 min, and their fluorescence intensity was assessed by fluorescence microscopy. The sample was concentrated as the buffer was continuously drained into the waste chamber. Therefore, the end point of scTFF was higher than that of the sample before filtration. As shown in Fig. 2(a), fluorescence intensity saturated at 2.5 h for DF and at 4.5 h for scTFF. The fluorescence intensity was nearly 1.5 times higher for scTFF than for DF, indicating that sample separation efficiency decreased over time for DF. Conversely, the fluorescence intensity of scTFF-separated samples increased continuously and became higher than that of the original sample, indicating concentration over time. Because the dcTFF system was designed for easy separation of heterogeneous samples without blocking the 200 nm membrane, we performed experiments in which a mixture of 50- and 500 nm PSBs was flowed through a chip, and recovery of PSBs collected in the sample chamber (C1) and isolation chamber (C2) was compared between scTFF and dcTFF. However, the operating fluid path for isolation of beads between scTFF and dcTFF is different because the dcTFF system has an additional bypass flow path to the sample chamber before entering the isolation chamber. Therefore, we compared the fluorescence intensity of PSBs obtained at C1 and C2 at the end point rather than comparing them over time. We then calculated the recovery rate of 50- and 500 nm PSBs according to the following equation: recovery rate = [C2/(C1 + C2)]. As shown in Fig. 2(b), all 500 nm PSBs were all filtered through the 200-nm membrane in C1 in both scTFF and dcTFF; thus, there was no significant difference in recovery of 500-nm PSBs between scTFF and dcTFF. On the other hand, the fluorescence intensity of 50 nm PSBs was nearly 1.8 times higher for scTFF than for dcTFF, indicating that the separation yield of 50 nm PSBs is increased with dcTFF because fluid flow is continuous from the sample chamber without interference of 500 nm PSBs with 50 nm PSBs. To validate that our dcTFF chip separated PSBs to the appropriate outlet, we injected 500- and 50 nm PSBs into the dcTFF chip [Figs. 2(c) and 2(d)]. Under these conditions, 500 nm PSBs were isolated under the 200 nm pore size membrane in C1 [Fig. 2(c)] and 50 nm PSBs were isolated under the 30 nm pore size membrane in C2, as confirmed by SEM [Fig. 2(d)]. A comparison of the permeation effectiveness of DF and scTFF, determined by measuring PSB fluorescence intensity, revealed that permeated 50 nm PSBs could attach to the second filter containing the 30 nm membrane surface in the DF system, adversely affecting their subsequent passage through the 200 nm membrane. In contrast, the scTFF system was less affected by the second filter containing the 30 nm membrane by virtue of the included circulation feature. Importantly, because the bypass path can reduce the transmembrane pressure in the dcTFF chip, the filtered yield for heterogeneous samples was higher for dcTFF than for scTFF, as evidenced by the observed clogging of the 200 nm membrane with 50 nm PSBs in the scTFF system but not the dcTFF system. Collectively, these findings confirm that the dcTFF system overcomes clogging effects and is thus able to isolate EVs within a specific filtered size range between the two membranes.

### Evaluation of filtration performance for EV isolation

We next evaluated differences in the physical properties of EVs obtained from B16BL6 cell culture media using DF, scTFF, and dcTFF systems by characterizing their size distribution (by DLS) and morphology (by SEM). EVs remaining in C1, C2, and C3 chambers separated by DF, scTFF, and dcTFF were measured three times using DLS analysis, respectively.

**FIG. 3.**
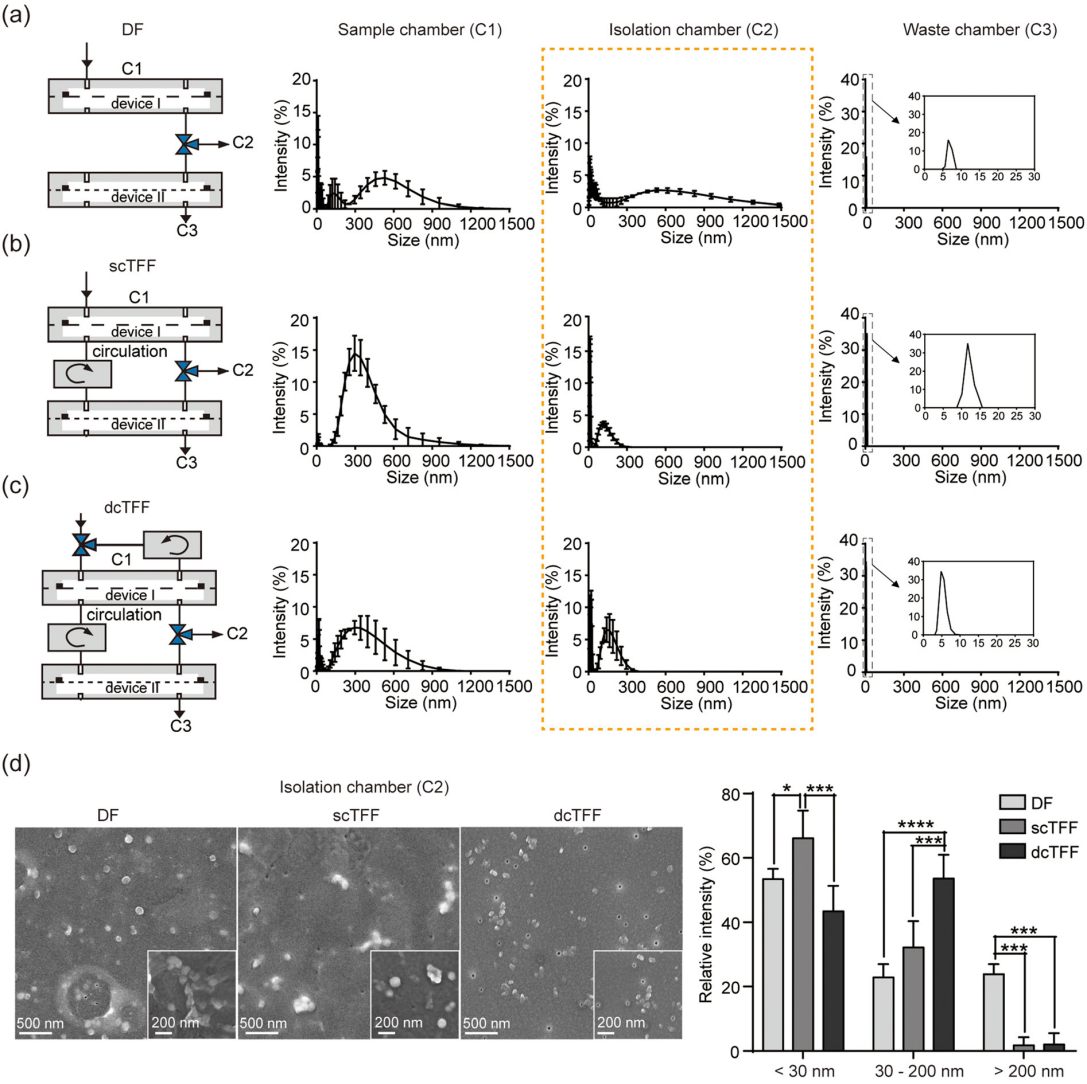
Comparison of the performance of DF, scTFF, and dcTFF in filtering EVs from B16BL6 cell media. (a)–(c) Schematics of DF (a), scTFF (b), and dcTFF (c) showing inlet and outlet positions and DLS measurements of the size distribution of EVs using DF, scTFF, and dcTFF filtration. (d) SEM images of EVs on a 30 nm membrane isolated by DF, scTFF, and dcTFF, and a comparison of EVs in C2 [yellow dotted line of (a)–(c)] within a specific measured size range isolated by DF, scTFF, and dcTFF. Data are presented as means ± standard error of mean (SEM) (^*^ *p *<* *0.05, ^***^ *p *<* *0.001, ^****^ *p *<* *0.0001).

As shown in Figs. 3(a)–3(c), a DLS analysis of C1 yielded a wide range of estimated sizes for DF, scTFF, and dcTFF systems, whereas an analysis of C3 yielded an estimated particle size less 10 nm in all cases. DLS analysis of C2 yielded a broad range of estimated sizes in DF; however, for scTFF and dcTFF, EVs of less than 300 nm were mainly observed in C2. In addition, the intensity of EVs was relatively higher in dcTFF than in scTFF. For scTFF and dcTFF, the average particle size is 150 nm and the smallest particle size is 10 nm.

An SEM characterization of the morphology of EVs isolated from cell culture media revealed that isolated EVs were different between DF, scTFF, and dcTFF [Fig. 3(d)]. An image of the 30 nm membrane showed aggregation and clogging of EVs using the DF chip, with traces of air bubble generation. Differences among DF, scTFF, and dcTFF systems were compared by analyzing the size range of EVs isolated between 30- and 200 nm membranes (C2) [Fig. 3(d)]. A histogram of modal EV size in C2 for DF showed sizes generally smaller than 30 nm and larger than 200 nm. On the other hand, histograms of modal EV size for scTFF and dcTFF revealed a decrease in EVs with sizes larger than 200 nm. This indicates that the DF process, operating at constant pressure, causes aggregation and clogging of EVs measuring larger than 200 nm. EVs with sizes smaller than 200 nm were increased by the scTFF process, with the device II operating circulation system, and were further increased by the dcTFF process, with both device I and II operating circulation systems together. In a cyclic TFF system, because the sample fluid flows continuously parallel to the membrane and not perpendicular to the membrane and the circulation flow rate is four times higher than the injection flow rate, it is less likely that the EVs stick to the device or membrane or transform into a smaller EVs due to a pressure drop.

### Dual cyclic tangential flow filtration device for EV isolation

**FIG. 4.**
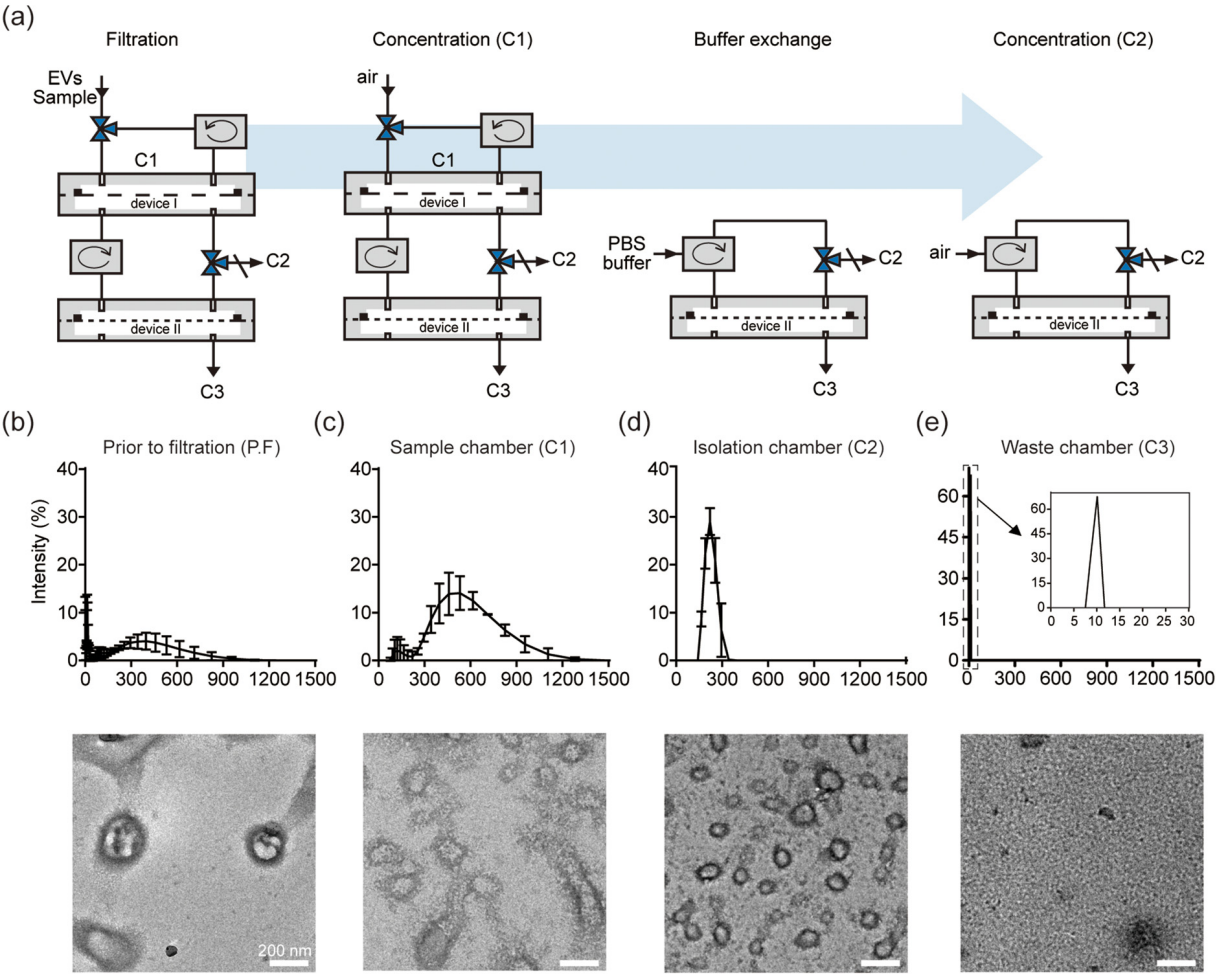
Evaluation of EVs isolated from B16BL6 cell media using dcTFF. (a) Schematic of dcTFF showing operation of the circulation system process. (b)–(e) DLS measurements of size distribution and TEM images of EVs at each size cutoff from B16BL6 cell culture media prior to filtration (b), from sample chamber C1 (c), from isolation chamber C2 (d), and from waste chamber C3 (e).

To this point, our data indicate that the dcTFF process improves the effective separation of a biological sample into a specific size range through dual filtration without blockage of the filter. However, we found that EVs with a size smaller than 30 nm still remained in C2 [Fig. 3(c)]. Further investigation of the morphology and size of EVs by TEM showed that filtered EVs were not evident by TEM owing to contaminants (Fig. S2). To remove contaminants, we added PBS and, after filtration, flowed air through the device to concentrate isolated EVs at C2 [Fig. 4(a)]. Specifically, after injection of culture media into the system, we separated device I and device II and performed buffer exchange by injecting PBS into device II containing EVs with a size smaller than 200 nm. We then measured the size of isolated particles at each outlet. As shown in Figs. 4(b)–4(e), prior to filtration, EV sizes were spread over a wide range, from 1 nm to 1000 nm, and few EVs were detected by TEM per field of view [Fig. 4(b)], whereas the size distribution of particles in C1 was larger than 200 nm, as measured DLS [Fig. 4(c)]. The hydrodynamic diameters of EVs in C2, measured by DLS, were approximately 150 nm and were close to circular in shape with a diameter of approximately 100 nm in TEM images [Fig. 4(d)]. EVs in C3 measured approximately 10 nm in diameter and corresponded to waste products obtained during the concentration of EVs [Fig. 4(e)]. Taken together, size distribution histogram data show that different sizes appeared in each outlet; moreover, results obtained by TEM were consistent with those obtained by DLS. We found that EVs with a size larger than 30 nm and smaller than 200 nm were increased by the dcTFF process operating circulation system devices I and II together. This indicates that the separation yield of EVs was increased by operating the device II circulation system to eliminate soluble protein in C2 and by the absence of larger vesicles interfering with device I EVs in C1.^26^ This suggests that EVs were simultaneously collected and concentrated in the isolation chamber during dual continuous flow conditions and that most EVs obtained were within the specific filtered size range of 30–200 nm.

**FIG. 5.**
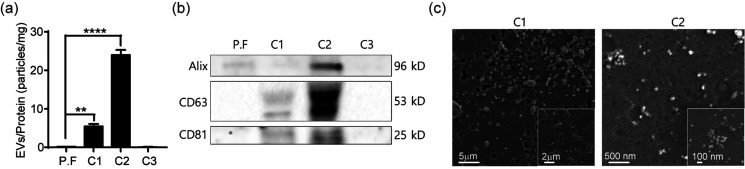
Characterization of cancer cell-derived EVs isolated by dcTFF. (a) Purity ratio of EVs isolated by dcTFF. (b) Western blot analysis for Alix, CD63, and CD81 in EVs isolated by dcTFF. (c) SEM images of EVs on 200 nm and 30 nm membranes isolated by dcTFF. Data are presented as means ± standard error of mean (SEM) (^**^ *p *<* *0.01, ^****^ *p *<* *0.0001).

We assessed the purity of exosomes by evaluating the number of EVs per field of view in TEM and by measuring the protein concentration of particles isolated from cell culture media at each outlet, as described in Methods (Fig. S3).^27^ The highest protein concentration was for particles isolated in C3, whereas the lowest was for particles isolated in C2 [Fig. 5(a)]. Nevertheless, the EV/protein ratio for C2 (24 particles/mg of protein) was higher than that for other outlets. By comparison, the EV/protein ratio obtained prior to filtration was 0.2 particles/mg, indicating highly efficient concentration of EVs from cell culture media. We also confirmed the presence of the exosome protein biomarkers, Alix, CD63, and CD81 in particles isolated in C2 [Fig. 5(b)], and characterized EVs isolated from cell culture media using SEM [Fig. 5(c)].^28^ Additionally, we evaluated our ability to isolate EVs from volumes of samples as small as 0.5 ml. Figure S4 shows that the recovery rate from 1 ml was similar to that from 10 ml, but the recovery rate from 0.5 ml samples was reduced to 0.6-fold. Since the working volume of dcTFF including tubing is about 1 ml, loss may occur during the isolation process at lower volumes.

### Evaluation of EV isolation by dcTFF and ExoQuick

**FIG. 6.**
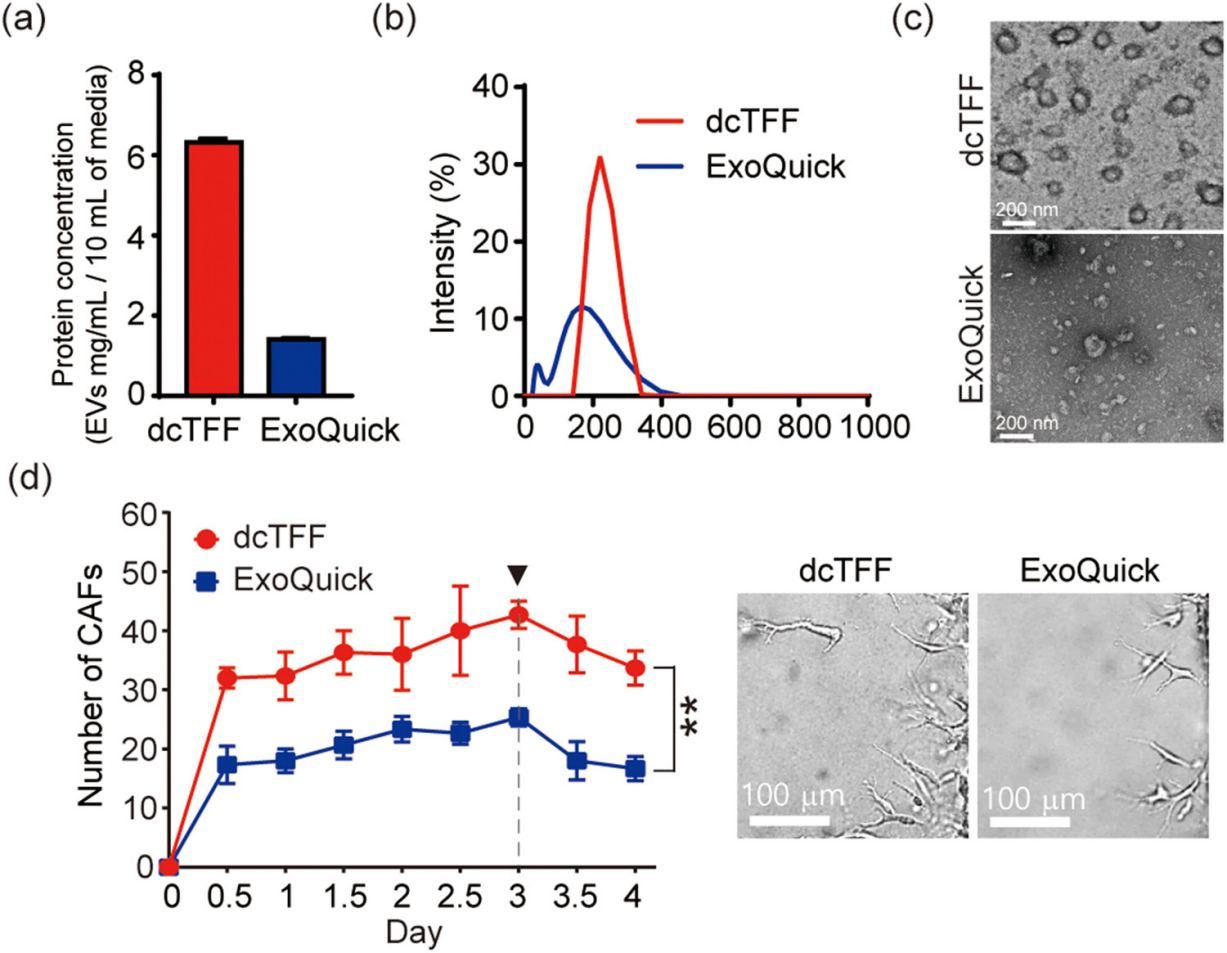
Comparison of cancer cell-derived EVs isolated by dcTFF and Exoquick. (a) Evaluation of protein concentration of EVs. (b) Size distribution and z-average of EVs. (c) TEM images of EVs. (d) Verification of EV activity that induces differentiation of cancer-associated fibroblasts (CAFs). Data are presented as means ± standard error of mean (SEM) (^**^ p < 0.01).

Finally, we compared EV isolation from cell culture media using the dcTFF chip and the conventional ExoQuick EV isolation technique, characterizing the quantity and morphology of isolated EVs. An analysis of protein concentration of isolated EVs revealed that the yield of EVs obtained using the dcTFF chip was 4.5-fold higher than that using ExoQuick starting from the same volume of media (10 ml) [Fig. 6(a)]. The size distribution of EVs obtained by ExoQuick alone ranged from ∼20 to ∼400 nm and showed a more discrete distribution of peaks than dcTFF alone [Fig. 6(b)]. Notably, the dcTFF chip resulted in more uniform EVs than ExoQuick, as evidenced by a more discrete size distribution [Fig. 6(b)]. Additionally, TEM revealed that EVs isolated using ExoQuick exhibited a spherical morphology and a wide size range. Furthermore, we compared the size of EVs separated by dcTFF and ultracentrifugation. The ultracentrifuged EVs showed a wide size range from 50 nm to 1000 nm, while the EVs separated by dcTFF showed a unimodal distribution near the 300 nm size (Fig. S5). These results suggest that the various filtration methods tested here could be used to obtain desired EV sizes, depending on the purpose of isolation.

In Fig. S6, we compared the EVs isolated by dcTFF and ExoQuick using CD63 as a molecular marker for EVs and Annexin V as a marker for microvesicles.^29^ As a result, EVs isolated from dcTFF had a CD63 positive EVs that was 1.3 times higher than EVs separated by ExoQuick. In addition, EVs separated by ExoQuick had more Annexin V positive vesicles than EVs isolated by dcTFF. In conclusion, for EVs separated by dcTFF, the ratio of CD63 to Annexin V was 70 times higher than for EVs isolated by ExoQuick. In order to analyze the characteristics according to the specific types of isolated particles, we intend to perform density gradient classification in the future.^30^

To confirm that the cargo inside the exosomes was still active, cancer cell-derived EVs isolated by dcTFF and ExoQuick were delivered to HUVECs (human umbilical vein endothelial cells) to see if HUVECs differentiate into cancer-associated fibroblasts (CAFs). In the previous study, we have shown that when the genetic materials inside cancer cell-derived EVs delivered to HUVECs, HUVECs differentiated into CAFs through endothelial-mesenchymal transition (EndMT).^31^ As a result, both of the cancer-derived exosomes isolated by dcTFF and ExoQuick differentiated HUVECs into CAFs, but in particular, EVs isolated by dcTFF induced the differentiation of CAFs more aggressively than EVs isolated by ExoQuick.

Our findings indicate that a dual-cyclic TFF chip (dcTFF) effectively performs size-based EV sorting from a heterogeneous EV population and shows higher isolation efficiency compared with direct filtration (DF) and a single-cyclic TFF (scTFF) chip. The separated EVs can be sorted from cell culture media into different-sized EV subpopulations at a particular filter size in the appropriate outlet. The ability to separate EVs has potential application in studying cell lines for the modal size distribution and size of EVs within a specific size range and for comparative evaluation of EV marker proteins. Moreover, isolated EVs constitute noninvasive liquid biopsy samples that can provide comprehensive information about the original tumor.^32^ Accordingly, EV-based diagnostics can improve treatment guidance for therapy responses, predicting and monitoring drug resistance and prognosis over time.^10,33^

## CONCLUSIONS

We developed a completely enclosed, dual-cyclic TFF chip (dcTFF) designed using simple prototyping methods, such as acrylic components and an adhesive-free rubber-based approach for preventing fluid leakage. The complete dcTFF system employed here utilized 200 nm and 30 nm filter membranes for size-exclusion–based exosome isolation together with device I and II circulation systems. This integrated dcTFF chip platform was validated using 500 nm and 50 nm PSBs and was shown to exhibit higher separation efficiency of samples than DF or scTFF based on fluorescence intensity and SEM images. Our data suggest that the dcTFF chip can be used for filtration, buffer exchange, concentration, and elimination of soluble protein, without interference from larger vesicles, in one step. In addition to this, if a third cyclic pump could be added to the C3, it is expected that it will be less affected by the pressure applied to the 30 nm membrane due to the circulation effect, and it would help to diffuse out less than 30 nm particles. Furthermore, the ability to isolate EVs from sample volumes as small as 1 ml has potential diagnostic applications. In the future, we plan to test our system with real patient serum for diagnostic purpose.^34^ Additionally, we will seek to develop a multi-channel dual-filtration system for simultaneous detection of EVs from small volumes that would be practical in a clinical setting. This would be important for future biomedical applications and could be applied to high-throughput filtering for various clinical or research applications. EV-based diagnostics can help provide treatment guidance for optimal therapeutic responses, monitoring of drug resistance, and prognosis over time.

## METHODS

There are no experiments on human or animals in this study; therefore, ethics approval is not required.

### Device fabrication

Devices were assembled with the aid of a laser cutter. The dual-filtration device, with outer dimensions of 40 × 40 × 8 mm, was assembled using two layers of acrylic plates and two filter membranes [Fig. 1(a)]. Two circular chambers, 23 mm in diameter, 500 *μ*m in height, each with a volume of 157 *μ*l, were positioned above and below the membrane. Whatman Nuclepore track-etched membranes (GE Healthcare Life Science, NJ, USA) with pore sizes of 30 and 200 nm were assembled between two layers of acrylic plates in separate modules and sealed with a rubber gasket to prevent fluid from leaking and entering parts of the membrane outside of the filtering area. Each assembled dcTFF chip was connected through a silicon tube (Cole-Parmer, IL, USA) with an inner diameter of 0.5 mm [Fig. 1(b)]. Fluorescent polystyrene beads (PSBs) with diameters of 50 and 500 nm (Nanocs Inc., NY, USA) were used for validation experiments.

### Cell culture

B16BL6 murine melanoma cells [KCLB No. 8006; Korean Cell Line Bank (KCLB), Seoul, Korea] were plated on culture dishes and maintained in Minimum Essential Medium alpha (α-MEM; Gibco BRL, MD, USA) supplemented with 10% [v/v] fetal bovine serum (FBS; Rocky Mountain Biologicals, Missoula, MT, USA), 1% [v/v] penicillin and streptomycin (Lonza, Basel, Switzerland). All cells were incubated at 37 °C in a humidified 5% CO_2_ environment. Before detachment of cells from culture dishes, the culture medium was replaced with Dulbecco's Modified Eagle Medium (DMEM) supplemented with 10% exosome-depleted FBS (dFBS; System Biosciences, CA, USA) and 1% [v/v] penicillin-streptomycin to remove interference from FBS exosomes in media.

### Pre-processing step before separation of EVs

The cells were cultured with dFBS for 24 h and centrifuged at 500 × g for 10 min to remove cell debris. The collected supernatant was transferred to a new flask and re-centrifuged at 5000 × g for 30 min. After the final collection, the supernatant was centrifuged at 10 000 × g for 30 min. 10 ml of cell culture supernatant was connected to the tube of the device using a syringe and performed at 4 °C.

### Dynamic light scattering (DLS) analysis

Cancer cell-derived exosomes were characterized in terms of hydrodynamic size. The hydrodynamic size was evaluated based on size distribution and z-average of exosomes, determined with a Zetasizer Nano ZS90 system using the included scattered intensity autocorrelation functions (Malvern Instruments, UK).

### Protein quantification

The concentration of protein associated with EVs isolated from cell culture media was estimated using a Pierce bicinchoninic acid (BCA) protein assay kit (Thermo-Fisher Scientific, IL, USA). A standard curve was prepared by mixing 10 *μ*l of BCA standard solution with 200 *μ*l of BCA working solution in a 96-well plate, followed by incubation at 37 °C for 30 min. Absorbance was measured at a wavelength of 562 nm using a BioTek ELx800 microplate reader (BioTek, VT, USA).

### Scanning electron microscopy (SEM)

Filtering efficiency was analyzed before and after filtering by SEM imaging. Each membrane was cut into a 1 × 1 cm size and attached to a 51 mm circular stub using dual-sided carbon tape. Before loading prepared samples into the SEM chamber, the sample stub was coated using an osmium coater to prevent charge accumulation during SEM imaging. The morphology of membranes was observed using an SU 5000 SEM system (Hitachi, Japan) at accelerating voltages ranging from 1 to 10 kV.

### Transmission electron microscopy (TEM)

For TEM sample preparation, 4 *μ*l of sample solutions consisting of EVs isolated from B16BL6 cell culture media using dcTFF or an ExoQuick-TC kit (System Biosciences, CA, USA) were loaded onto carbon-coated 200-mesh cooper grids, surface-treated with oxygen plasma. Sample solutions were incubated on the grid for 1 min at room temperature to enhance adsorption of the sample to the carbon film. After incubation, the grids were washed three times with 20 *μ*l of de-ionized (DI) water and stained with 100 *μ*l of 2% (w/v) uranyl acetate solution. Excess staining solution was wicked away with Whatman filter paper (GE Healthcare Life Science), and the grids were air-dried for 10 min. The dried grids were observed using an ARM 200CF system (JEOL, Tokyo, Japan) equipped with a field emission gun and charge coupled device camera (USC 1000; Gatan Inc., CA, USA). An acceleration voltage of 200 kV was used.

### Western blot analysis

Exosome preparations, normalized to 20 μg protein content, were mixed with 5× Laemmli sample buffer (Elpisbio, Daejeon, Korea) and boiled for 5 min at 95 °C. Exosome proteins were resolved by sodium dodecyl sulfate polyacrylamide gel electrophoresis (SDS-PAGE) on 4%–20% gradient polyacrylamide gels (Cat# 456–1093; Bio-Rad, CA, USA) and subsequently transferred to a nitrocellulose membrane. After blocking with 5% skimmed milk solution in 1× Tris-buffered saline containing 0.1% (v/v) Tween 20 (TBST) for 1 h, the membrane was incubated overnight at 4 °C with anti-CD63 (1:1000, bs-1523; Bioss, MA, USA), anti-Alix (1:1000, ab88388; Abcam, Cambridge, UK), anti-CD81 (1:1000, bs-6943R; Bioss, MA, USA) and anti-Annexin V (1:1000, SC-8300; Santa Cruz Biotechnology, CA, USA) primary antibodies. The membrane was washed 5 times in 1× TBST for 1 h at room temperature (RT) and then incubated for 1 h at room temperature with horseradish peroxidase (HRP)-conjugated anti-rabbit IgG secondary antibody (1:2000 in TBST containing 5% skim milk; Cell Signaling Technology, MA, USA). After washing five times in 1× TBST for 1 h at room temperature, immunoreactive bands were detected using the Clarity Western Enhanced Chemiluminescence (ECL) kit (Bio-Rad, Cat #: 170–500) and Chemidoc imaging system (Bio-Rad).

### EV isolation using an ExoQuick-TC kit

For isolation of EVs using a commercially available method, ExoQuick-TC solution was added to the collected supernatant at a ratio of 1:5. The mixture was then shaken to homogeneity and stored at 4 °C for at least 12 h. After incubation, the mixture was centrifuged at 1500 × g for 30 min, yielding an EV pellet. The supernatant was aspirated and the remaining mixture was collected and centrifuged at 1500 × g for 5 min, leaving a white pellet at the bottom of the tube. The pellet was resuspended in 300 *μ*l of phosphate-buffered saline (PBS).

### Verification of EVs activity by inducing cancer-associated fibroblasts (CAFs) differentiation

To confirm the activity of EVs isolated by dcTFF and ExoQuick, we observed the differentiation of HUVECs and CAFs by cancer cell-derived EVs using a 3D microfluidic device developed in our previous study. We cultured HUVECs (CC-2517; Lonza, Basel, Switzerland) in the microfluidic device and then injected cancer cell-derived EVs isolated by dcTFF and ExoQuick from B16BL6 murine melanoma cell media, respectively. In addition, the flow direction of interstitial fluid was created through the microfluidic channel to induce differentiation from HUVECs to CAFs.^31,35,36^

## SUPPLEMENTARY MATERIAL

See the supplementary material for the comparison of filtration performance. We supplemented additional results for supporting dcTFF filtration performance.

## AUTHORS' CONTRIBUTIONS

K.K., H.H., and J.H.Y. conceived the hypotheses, methods, and application; K.K., J.P., J.-H.J., and R.L., performed experiments; K.K and J.H.Y. analyzed data; K.K., H.H., and J.H.Y. wrote the manuscript; and all authors discussed results and commented on the manuscript. J.-H. P. and J.M.Y. helped shape the research. All authors have read and agreed to the published version of the manuscript.

## Data Availability

The data that support the findings of this study are available from the corresponding author upon reasonable request.
